# Healthy Food, Healthy Teeth: A Formative Study to Assess Knowledge of Foods for Oral Health in Children and Adults

**DOI:** 10.3390/nu14142984

**Published:** 2022-07-21

**Authors:** Sarah Hancock, Grant Schofield, Caryn Zinn

**Affiliations:** The Human Potential Centre, Auckland University of Technology, P.O. Box 92006, Auckland 1142, New Zealand; grant.schofield@aut.ac.nz (G.S.); caryn.zinn@aut.ac.nz (C.Z.)

**Keywords:** dental diseases, dental caries, oral health, oral nutrition, nutrition knowledge, eating behaviour, processed foods, carbohydrates, health promotion, dietary guidelines, health education

## Abstract

Eating patterns characterised by low intakes of processed carbohydrates and higher intakes of fat- and Vitamin D-rich foods are associated with protection against dental caries. The aim of this formative study was to evaluate the extent to which the knowledge of children and adults of foods for oral health reflects dietary guideline advice, and the evidence base for foods associated with increased and decreased caries burdens. Using a novel card-sorting task, the participants categorised foods according to their knowledge of each food for oral health. There were no differences between children and adults in the categorisation of fresh, minimally processed foods. Fish, chicken, and red meat were categorised as healthy by significantly fewer children than adults. High-sugar foods were correctly characterised as unhealthy by nearly all participants. More children categorised breakfast cereals as healthy than adults. There were no statistically significant differences between children and adults for the categorisation of brown or wholegrain breads categorised as healthy. The alignment of the participants’ beliefs with dietary guideline recommendations suggests education through health promotion initiatives is successful in achieving knowledge acquisition in children and adults. However, recommendations to increase the intake of refined carbohydrates inadvertently advocate foods associated with increased caries burdens.

## 1. Introduction

Dental caries, a preventable disease of dietary origin, is the most common chronic childhood disease in New Zealand. There is increasing recognition of the effect of poor oral health on general health. Dental caries has dietary risk factors in common with obesity, and chronic disease for which hyperinsulinaemia and associated metabolic syndrome are implicated [1,2,3], including Type 2 diabetes [4] and cardiovascular disease [5,6]. The endpoint of oral disease is typically when people are likely to seek dental care, often with presentation of a cavitated carious lesion causing pain, discomfort, and/or infection [7,8].

The common dietary risk factor for caries and other chronic diseases is the high and frequent consumption of fermentable carbohydrates, particularly ultra-processed foods which have high amounts of refined starches and free sugars and are associated with eating patterns that exceed recommended sugar consumption levels of less than 10% of total energy intake [9,10,11,12]. Upon ingestion of these foods, endogenous bacteria in the biofilms produce organic acids as a by-product of the metabolism of fermentable carbohydrates [13,14,15]. These lactic, formic, and acetic acids cause local pH values in the dental biofilm to fall below neutrality to critical values of 5.5 and below, which results in the demineralisation of tooth tissues. Slow salivary clearance rates of high starch-containing foods and a resultant increased retention time in the mouth of these foods means the effects of other simultaneously present sugars may also be prolonged, where starch consumption has a co-cariogenic effect with other sugars [16,17,18]. A recent systematic review of prospective cohort studies found that between-meal consumption of processed sugar- and starch-containing foods was consistently found to be associated with greater caries experience [19].

Food intake can enhance enamel remineralisation when the diet contains sufficient vitamin D, calcium, and phosphates [20,21,22,23]. A vitamin D-rich diet has long been associated with reduced rates of dental caries [22,24,25,26]. The consumption of cheese is associated with protection from caries [20,27] with proposed mechanisms of protection including increased salivary flow and buffering, inhibition of plaque bacteria overgrowth, and through the intake of calcium, inorganic phosphate and casein, which enhance remineralization [28]. In addition, carious lesions may be arrested by remineralisation at initial pre-cavitation stages by dietary intervention with Vitamin D-rich foods [24].

Nutrition education is one of several components of intervention strategies for health improvement [29,30]. To develop educational interventions around dietary lifestyle change to prevent caries in children and their families, it is imperative to understand the prior food knowledge of children, and adults who would be involved in the design, implementation, and uptake of such interventions.

Studies of oral health knowledge in children have evaluated beliefs regarding foods in the context of oral health interventions occurring in school settings [31,32,33,34] and associations between dental pain and intake of particular foods [35]. The study of oral health nutrition knowledge in parents has typically evaluated the knowledge of parents and caregivers in relation to the caries status of their children [36,37,38]. Inverse relationships between the oral health knowledge of parents regarding oral hygiene practices and nutrition and the presence of caries in children have been observed in these studies.

Oral health food knowledge has been studied in a range of health professionals [39,40,41,42,43,44,45,46]. The classification of foods as healthy or otherwise was in accordance with recommendations and advice outlined in government-endorsed dietary guidelines for healthy eating of the respective countries of study [42,47,48,49,50,51]. However, guidance provided in dietary guidelines regarding food intake may also not reflect the evidence of the aetiological causes of dental caries. In New Zealand, dietary advice for oral health reflects advice provided for general health in dietary guidelines for a population free of any chronic condition, but the recommendations provided in these guidelines contradict the evidence of dietary causes of dental caries [52].

The aim of the study was to evaluate the extent to which the knowledge of children, parents, and health professionals regarding foods for optimal oral health reflects advice provided in dietary guidelines for healthy eating, and the evidence base regarding foods for caries prevention. An additional objective was to assess any variation between these disparate groups of participants in how they categorised foods as healthy or unhealthy for oral health.

## 2. Materials and Methods

### 2.1. Recruitment

Principals of eight primary and intermediate schools in the Lake Taupō area in the central North Island of New Zealand were approached to have their respective schools involved as recruitment centres and study sites. Permission was granted from four schools to participate in the study.

On receipt of principals’ consent, children aged 10–13 years and the parents/caregivers of the children were recruited using a flyer distributed through the school’s usual communication method. This age group of children was selected because it was estimated they would have the cognitive skills necessary to complete the tasks, have been exposed to health promotion messages as part of the school curriculum for a minimum of five years, and would be likely to have more independence in choosing foods [53,54]. In addition, younger children have been shown to judge foods as healthy based on personal preference [55]. Children were eligible if they were between 10 and 13 years of age and were literate. Parents, similarly, were eligible if one of their children between 10 and 13 years of age attended any of the study schools and were literate. All parents who responded to the advertisement were screened for eligibility and provided written informed consent for their child to participate in the card sorting exercises. Parents were also invited to participate through the same methods as above and provided informed consent to participate in the study. Eligible children completed assent forms for participation and data use in the presence of a parent or a member of the teaching staff at the school. Health professionals including doctors, nurses, and dental professionals were also invited to participate through a general medicine practice, a dental clinic, and a community-based charitable trust. Budget, time, and resource constraints precluded further recruitment from other clinics and health service providers. All participants were reimbursed with a store voucher to the value of NZD10 in appreciation of their time. Ethical approval for this study was granted by the Auckland University of Technology Ethics Committee on 8 July 2018 (18/82).

### 2.2. Procedures

**Figure 1:**
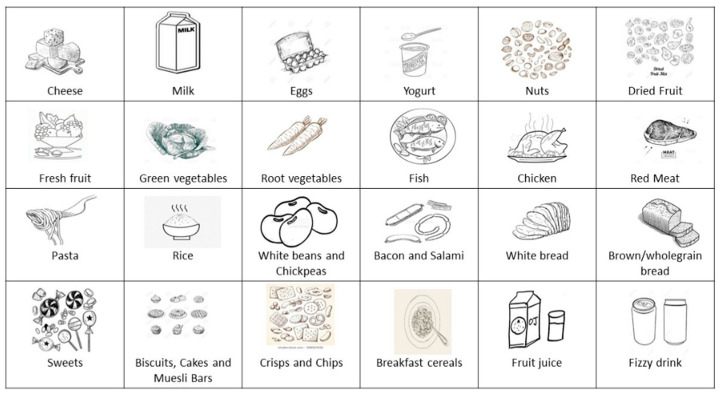
Picture cards of foods to be categorised by children, parents/caregivers, and health professionals.

All participants completed a picture-card sorting exercise in which 24 food cards were allocated to categories according to how they ranked each food based on their beliefs of whether it is “good” or “bad” for oral health. This method was used to enable comparisons to be made between children, parents, and health professionals. The foods chosen for the cards were derived from the New Zealand National Children’s Nutrition Survey [56]. The pictures of foods were royalty-free, black-and-white sketches obtained from Google Images as illustrated in Figure 1. The development of the card sorting tasks and the testing of validity and reliability have previously been outlined [57].

Photographs were taken of each participant’s cards allocated to the categories of “good” or bad” with their unique identifier number placed beside their cards; there was no photography or recording of identifiable characteristics of any of the participants. The data contained in the photographs were entered in a Microsoft Excel spreadsheet and stored on a password-protected computer. The frequency with which each food was reported in the categories pertaining to whether the food was “good” or bad” for oral health was tabulated.

### 2.3. Analyses

Demographic information regarding the age, gender, and ethnicity of all participants was recorded. Parents and health professionals provided additional information on employment type and highest educational attainment.

The data from the picture card sorting exercise were analysed according to correctness of foods for general health based on recommendations from the New Zealand Dietary Guidelines [58]. Foods classified as healthy according to these guidelines were fresh fruit, green vegetables, root vegetables, cheese, milk, unsweetened yoghurt, nuts, chicken, fish, red meat, white beans and chickpeas, rice, breakfast cereals, pasta, and brown or wholegrain bread. Foods classified as unhealthy were dried fruit, fruit juice, bacon and salami, white bread, fizzy drink, crisps and chips, sweets, and biscuits and cakes. Sugar-sweetened beverages are colloquially referred to in New Zealand as “fizzy drink”, particularly by children. This group of beverages does not include sparkling water or soda water.

Participants were awarded one point for each correct categorisation of a food, i.e., if they rated foods as either “healthy” or “unhealthy”. The maximum number of points that could be attained was 24 points. Differences between how children, parents, and health professionals rated the foods for oral health were assessed by calculating the mean differences in proportions (PD) of participants who correctly rated the foods as “good” or “bad” for oral health.

The proportions of correctly rated scores for each group were compared using the analysis of mean differences in proportions (PD) in Microsoft Excel™ of correctly rated foods between each group of participants.

## 3. Results

Informed consent was provided by parents and caregivers for 79 children aged between 10 and 13 years to participate in the study. The children also completed assent forms. Nine adults and twelve health professionals also provided informed consent for study participation. The reasons given by other schools for non-participation were staffing issues and ongoing construction work within the school environments. The demographic characteristics of the child and adult participants are presented in Table 1. 

### 3.1. Comparisons of Participant Groups Using the Card Sorting of Foods by the Current Dietary Guidelines Model

The mean correct total scores for children, parents, and health professionals are presented in Table 2.

The mean correct scores ranged from 76% of cards being correctly categorised out of a possible 24 cards for the child participants to 89% for the parents. There were no statistically significant differences observed between parents and health professionals (PD: 8%, 95% confidence interval (CI): −27–38%). For this reason, the results for all adults were combined for further analyses. The mean percentage of correctly categorised foods of all adult participants (84%) was slightly higher than that for the children (76%): this difference in proportions was not statistically significant (PD: 8%, 95% CI: −14–23%).

The maximum number of points that could be achieved was 24 points.

### 3.2. Categorisations of Foods by Children and Adults: Foods Recommended as Healthy According to Dietary Guidelines

**Figure 2:**
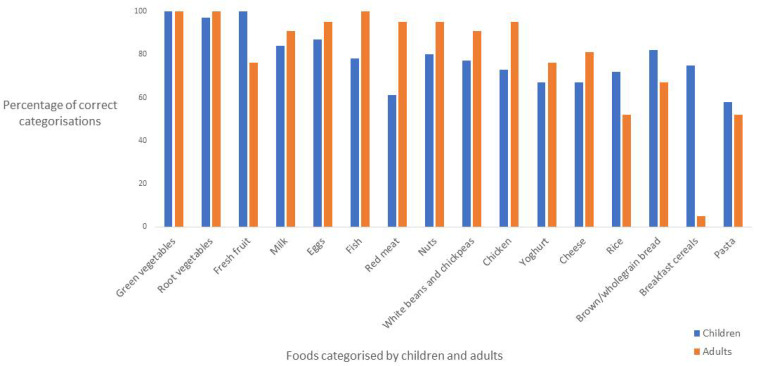
Categorisations of foods for oral health as “healthy” by adults (*n* = 21) and children (*n* = 79) using current dietary guidelines.

The proportions of child and adult participants who correctly categorised each food item as healthy according to recommendations based on the current dietary guidelines are presented in Figure 2.

There were some differences in how children and adults categorised other minimally processed foods that were regarded as “good” for oral health. Fresh fruit was classified as good for oral health by all children, and by three-quarters of adults; this difference was statistically significant (PD: 24%, 95% CI: 10–45%,). Most minimally processed foods were correctly categorised as healthy by most participants. All participants categorised green vegetables as “good” for oral health, and other foods including root vegetables, milk, eggs, nuts, and white beans and chickpeas were rated as good for oral health by nearly all participants. There were no statistically significant differences between the proportions of children and adults who regarded these foods as good for oral health.

Statistically significant proportion differences were observed in the categorisation of the following foods, where more adults categorised these as good for oral health compared to children: fish (PD: 22%, CI: 5–32%), chicken (PD: 22%, 95% CI: 2–33%), and red meat (PD: 34%, 95% CI: 13–46%).

There were no significant differences between the proportions of children and adults in the categorisation of brown and wholegrain bread and pasta, respectively, for oral health. Statistically significant differences were observed between the proportions of children and adults in the categorisation of breakfast cereals for oral health; slightly more than three-quarters of the children categorised breakfast cereals as healthy compared to one adult (PD: 70%, 95% CI: 49–79%).

### 3.3. Categorisations of Foods by Children and Adults: Foods Recommended as Unhealthy According to Dietary Guidelines

**Figure 3:**
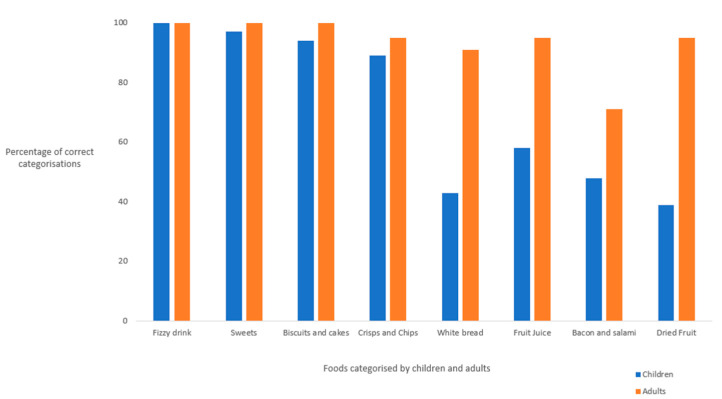
Categorisations of foods for oral health as “unhealthy” by adults and children (*n* = 21) and children (*n* = 79) using current dietary guidelines.

The proportions of child and adult participants who categorised each food item correctly as unhealthy according to recommendations based on the current dietary guidelines are presented in Figure 3.

Statistically significant differences were observed between adults and children for the correct categorizations of fruit juice (PD: 37%, 95% CI: 16.3–48.7%) and dried fruit (PD: 56%, 95% CI: 35–67%), each categorised as bad for oral health because of high sugar content. There were no statistically significant differences in the proportions of children and adults who categorised bacon and salami as harmful for oral health.

There were statistically significant differences in the proportions of children and adults who classified white bread as unhealthy: less than half of the children classified white bread as unhealthy, compared to nearly all adults (PD: 48%, 95% CI: 26–60%). High sugar foods including fizzy drink, sweets, biscuits and cakes, and crisps and chips were correctly classified as “bad” for oral health by nearly all adults and children. There were no statistically significant differences observed in the proportions of children and adults who correctly categorised each of these foods as detrimental for oral health.

## 4. Discussion

The principal findings of this study were that the beliefs regarding healthiness of foods in disparate groups of children, parents, and health professionals reflect recommendations provided in government-approved dietary guidelines for healthy eating in children and young people. Most participants correctly categorised fresh, unprocessed foods as beneficial for oral health and high-sugar foods as detrimental for oral health. Adults attained slightly higher scores than children for the correct categorisation of foods, but these differences were not statistically significant. There was limited and mixed understanding in children and adults, respectively, regarding the beneficial effects of animal produce including chicken, fish, and red meat for oral health and the detrimental effects of ultra-processed, carbohydrate-containing foods for oral health.

The use of a picture-card sorting exercise to evaluate oral health food knowledge was a relatively novel element of this study that enabled direct comparisons of oral health food knowledge to be made across three groups of children, parents, and health professionals. Gamification strategies have been used to improve nutritional knowledge about healthier nutritional habits in young people and as a tool to assess nutrition knowledge [59]. In a recent study by Walker et al., parents and health professionals expressed a desire for preventive nutrition interventions to include interactive content, gamification, and practical resources with which to translate knowledge to practice [60]. The participant burden of the card sorting exercise was minimal and acceptable to the participants with completion of the task achieved in approximately 15 min.

The findings from this study should be interpreted with some caution, given the formative nature of the research. The study was limited to a small area of the central North Island of New Zealand. Time, budget, and personnel constraints resulted in insufficient time to recruit further adults for this study. Of interest is a higher number of Māori child participants (41% of all child participants) compared to census information from 2018 showing that Māori comprise 29.9% of the Taupō population [61]. Although the findings of this research may be broadly applicable to other regions in New Zealand, there may be less applicability of the findings internationally, particularly in countries with vastly differing food, health, social, and political systems. The small number of children and adult participants did not permit the comparison of results by ethnic group. This would be useful in future research because New Zealand European, Māori, Asian, and Pacific peoples have substantially different dietary histories [62]. Further investigation using the card sorting tasks in a larger population sample would be useful to stratify the findings by age, gender, and ethnicity. The findings from the statistical testing of differences between health professionals and parents as well as adults and children should be interpreted with some caution because of the low numbers of adults who participated in the study and the consequent lack of statistical power. The scores attained by the adult participants were high; this may reflect higher levels of interest in nutrition and nutrition knowledge and may also be a reason why these participants chose to participate in this study. In addition, there was no recording of dietary data or oral health status of any participants for this study, which may have provided useful information regarding the extent to which dietary behaviours reflected beliefs about the healthiness or otherwise of individual foods.

Most participants correctly categorised unprocessed foods, including green and root vegetables and eggs, as beneficial for oral health. Children and adults also correctly categorised sugar and high-sugar products as harmful for oral health when assessing foods in the picture sorting exercise. The high food knowledge scores attained by the participants in this study, and the alignment of this knowledge with recommendations in dietary guidelines, suggest that the provision of advice through dietary guidelines and health promotion initiatives can achieve knowledge acquisition in these groups. These findings are similar to those of previous research where the classification of foods as healthy or otherwise was in accordance with recommendations outlined in government-endorsed dietary guidelines for healthy eating of the respective countries of study [42,47,48,49,50].

Children attained significantly lower scores than adults for the correct categorisation of fish, chicken, and red meat as beneficial for oral health. The lower scores for the latter two foods also reflect advice in dietary guidelines regarding fat consumption, with recommendations to eat lean, low-fat animal produce [52]. In addition, recommendations regarding caveats around the consumption of meat and dairy produce were also learned by children and adults through health promotion initiatives. However, the intake of vitamin D-rich foods such as meat and fish has long been associated with reduced risks of dental caries [22,24,26]. Further, the evidence relating to reductions in dental caries in children with higher intakes of full fat dairy products is not reflected in current dietary guidelines [52]. The disconnects between the beliefs identified in this study regarding foods that are important for oral health, advice provided in dietary guidelines, and the evidence regarding dietary causes of dental caries indicates that there are substantial limitations in oral health education for the prevention of caries through dietary intake of caries-protective foods.

Breakfast cereals were categorised by 75% of the child participants as at least “good” for oral health compared to 5% of the adult participants categorising breakfast cereals as good for oral health. This finding in adults may reflect the growing awareness in the general population of the high sugar content of cereal-based foods, including a range of hidden sugars. The “What the Fat? Fat’s IN, Sugar’s OUT” book [63] has sold approximately 150,000 copies, and documentaries such as “That Sugar Film” [64] have been popular in Australia and New Zealand. The categorisation of cereals by children and of brown and wholegrain breads by most participants as good for oral health aligns with advice in dietary guidelines. However, promoted foods within these guidelines include ultra-processed items such as breads and cereals, which are high processed sugar- and starch-containing foods, or in the case of porridge oats, typically consumed with added sugars [65,66,67]. This advice contradicts long-established evidence of the role of high carbohydrate foods in dental caries [52] and, in particular, evidence regarding the associations between consumption of processed sugar- and starch-containing foods and dental caries [68].

The findings from this formative study have a range of implications. Given the burden of dental caries in New Zealand, and the high personal and public costs of poor oral health, it is important and timely to consider dietary interventions to improve oral health and reduce the risk of other chronic diet-related disease in New Zealand families. To design such interventions, it is important to understand the prior beliefs and knowledge of nutrition concepts as they relate to health and dental care of populations who are the target of such interventions and health practitioners who instigate such interventions. The involvement of, and contribution by, groups for whom the intervention is targeted may increase the likelihood that the interventions will be feasible, acceptable, and sustainable for participants.

The nutrition knowledge and beliefs of disparate groups of children, parents, and health professionals aligned broadly with recommendations in New Zealand’s current dietary guidelines for healthy eating in children and young people; this knowledge extended to beliefs about foods including full fat dairy products and meat for which there are caveats for consumption in the guidelines. Recommendations in New Zealand’s government-endorsed dietary guidelines continue to focus on reducing intakes of single macronutrients such as saturated fat. The emerging evidence on the relationships between high consumption of ultra-processed food products and poor health outcomes should provide impetus to translate findings through more coherent nutritional guidelines that are based on foods. Food-based classifications are used increasingly in some countries to inform nutrition guidance; one such country is Brazil, where the NOVA classification scheme is used to classify foods according to levels of processing [69]. Terminology such as “minimally processed”, “processed”, and “ultra-processed” foods is used in the set of guidelines statements for the population [70].

There is increasing recognition that the consumption of a Western diet, characterised by large amounts of ultra-processed carbohydrate-based foods, is detrimental for health [71,72]. Methods of classifying foods according to benefit and harm for health include the NOVA classification system, by which foods are classified according to the extent and purpose of industrial processing [65,66,67]. Ultra-processed products according to this classification include a minimum of five ingredients including sugar, oils, fats, salt, antioxidants, stabilisers, and preservatives [67]. Other additives in these products include dyes, colour stabilisers, and non-sugar sweeteners, and substances that have been manufactured using techniques including carbonating, firming, extrusion, and moulding. Ultra-processed products are hyper-palatable, packaged attractively, highly profitable, have high health ratings according to algorithm-based rating systems, and are aggressively marketed to children and young people [65,66,67]. The displacement of minimally processed foods by ultra-processed foods is associated with unhealthy dietary nutrient profiles [65,73,74] and several diet-related chronic diseases [71]. The high and frequent consumption of breads, cereals, and other ultra-processed, carbohydrate-containing foods is also associated with dental caries [19]. It is imperative to establish uniform delivery of messages about the role of ultra-processed, sugar-and starch-containing foods in dental caries, and the caries-protective effects of eating full-fat dairy produce and vitamin D-rich foods.

There is also a lack of consumer understanding of the terminology around sugars in food; this is exacerbated by the presence of sugars in ultra-processed foods promoted for health benefits in current dietary guidelines [58], and the ubiquity of hyper-palatable, highly profitable ultra-processed foods in the food supply that are classified as healthy using star rating systems [75,76]. Current suggestions to “eat less sugars” in oral health promotion may therefore be too simplistic, given this lack of universal understanding. A solution for aligning recommendations in dietary guidelines with the evidence may be to adopt a food classification system, which would also allow greater consistency between the evidence of benefits in oral health and the consumption of minimally processed, full-fat, vitamin D-rich foods.

## 5. Conclusions

Dental caries in children and young people is an ongoing and significant public health challenge in New Zealand. The beliefs and knowledge of foods for oral health identified in this study of disparate groups of children, parents, and health professionals are broadly aligned with recommendations provided in dietary guideline advice used in oral health promotion initiatives. In addition, recommendations in such health promotion strategies appear to be absorbed and learned by children and adults, who are the targets of such initiatives. However, the high and frequent intake of ultra-processed carbohydrate-based foods recommended in the guidelines inadvertently results in the advocacy of eating patterns associated with dental caries, obesity, and poor metabolic health through the life course. It is imperative that the guidelines for healthy eating be updated using a paradigm relating to the processing of foods, given the evidence of increased dental caries burdens alongside other chronic diseases associated with the consumption of ultra-processed food products.

## Figures and Tables

**Table 1:** Participant characteristics of the adults and children.

Participant Variable	Parents (*n* = 9) and Health Professionals (*n* = 12)
Age	
25–34 (%)	2 (10)
35–44 (%)	10 (48)
45–54 (%)	8 (38)
55–64 (%)	2 (10)
Gender	
Male (%)	6 (29)
Female (%)	15 (71)
Ethnic group	
NZ* European (%)	17 (81)
Māori (%)	1 (5)
Other (%)	3 (14)
**Participant Variable**	**Children (*n* = 79)**
**Age**	
10 years (%)	27 (34)
11 years (%)	32 (41)
12 years (%)	17 (22)
13 years (%)	3 (3)
**Gender**	
Male (%)	29 (37)
Female (%)	50 (63)
**Ethnic group**	
NZ * European (%)	43 (54)
Māori (%)	32 (41)
Asian (%)	3 (3.7)
Pacific (%)	1(1.3)

* New Zealand.

**Table 2:** Mean scores of correctness using current dietary guidelines for foods for oral health.

Participant Group	Mean Total Scores ± SD (Range)	Mean Percentage of Correct Total Scores (Range)
All Adults (*n* = 21)	20.2 ± 2.49 (12–24)	84 (50–100)
All Health Professionals (n = 12)	19.4 ± 2.91 (12–24)	81 (50–100)
All Parents (n = 9)	21.3 ± 1.22 (19–23)	89 (79–96)
All Children (*n* = 79)	18.3 ± 2.76 (11–23)	76 (46–96)

## Data Availability

The data presented in this study are available upon request from the corresponding author. The data are not publicly available due to confidentiality reasons.

## References

[1] Alrabiah M., Al-Aali K.A., Al-Sowygh Z.H., Binmahfooz A.M., Mokeem S.A., Abduljabbar T. (2018). Association of advanced glycation end products with peri-implant inflammation in prediabetes and type 2 diabetes mellitus patients. Clin. Implant Dent. Relat. Res..

[2] Negrini T.C., Carlos I.Z., Duque C., Caiaffa K.S., Arthur R.A. (2021). Interplay Among the Oral Microbiome, Oral Cavity Conditions, the Host Immune Response, Diabetes Mellitus, and Its Associated-Risk Factors-An Overview. Front. Oral Health.

[3] Liccardo D., Cannavo A., Spagnuolo G., Ferrara N., Cittadini A., Rengo C., Rengo G. (2019). Periodontal Disease: A Risk Factor for Diabetes and Cardiovascular Disease. Int. J. Mol. Sci..

[4] Chopra A., Jayasinghe T.N., Eberhard J. (2022). Are Inflamed Periodontal Tissues Endogenous Source of Advanced Glycation End-Products (AGEs) in Individuals with and without Diabetes Mellitus? A Systematic Review. Biomolecules.

[5] King S., Chow C.K., Eberhard J. (2022). Oral health and cardiometabolic disease: Understanding the relationship. Intern. Med. J..

[6] Rahimi A., Afshari Z. (2021). Periodontitis and cardiovascular disease: A literature review. ARYA Atheroscler.

[7] Jatrana S., Crampton P., Filoche S. (2009). The case for integrating oral health into primary health care. N. Z. Med. J..

[8] Vernon L.T., Teng K.A., Kaelber D.C., Heintschel G.P., Nelson S. (2021). Time to integrate oral health screening into medicine? A survey of primary care providers of older adults and an evidence-based rationale for integration. Gerodontology.

[9] Sheiham A., James W.P. (2015). Diet and Dental Caries: The Pivotal Role of Free Sugars Reemphasized. J. Dent. Res..

[10] Sheiham A., James W.P. (2014). A reappraisal of the quantitative relationship between sugar intake and dental caries: The need for new criteria for developing goals for sugar intake. BMC Public Health.

[11] Moynihan P., Tanner L., Holmes R., Hillier-Brown F., Mashayekhi A., Kelly S., Craig D. (2019). Systematic Review of Evidence Pertaining to Factors That Modify Risk of Early Childhood Caries. JDR Clin. Trans. Res..

[12] Moynihan P., Miller C. (2020). Beyond the Chair: Public Health and Governmental Measures to Tackle Sugar. J. Dent. Res..

[13] Loesche W.J. (1986). Role of Streptococcus mutans in human dental decay. Microbiol. Rev..

[14] Loesche W.J. (1997). Association of the oral flora with important medical diseases. Curr. Opin. Periodontol..

[15] OmerOglou E., Karaca B., Kibar H. (2022). The role of microbiota-derived postbiotic mediators on biofilm formation and quorum sensing-mediated virulence of Streptococcus mutans: A perspective on preventing dental caries. Microb. Pathog..

[16] Kashket S., Yaskell T., Murphy J.E. (1994). Delayed effect of wheat starch in foods on the intraoral demineralization of enamel. Caries Res..

[17] Linke H., Birkenfeld L. (1999). Clearance and metabolism of starch foods in the oral cavity. Ann. Nutr. Metab..

[18] de Sousa E.T., Lima-Holanda A.T., Sales L.S., Nobre-Dos-Santos M. (2021). Combined effect of starch and sucrose on carbonic anhydrase VI activity in saliva and biofilm of children with early childhood caries. Exposure to starch and sucrose alters carbonic anhydrase VI activity in saliva and biofilm. Clin. Oral Investig..

[19] Hancock S., Zinn C., Schofield G. (2020). The consumption of processed sugar- and starch-containing foods, and dental caries: A systematic review. Eur. J. Oral Sci..

[20] Kashket S., DePaola D.P. (2002). Cheese consumption and the development and progression of dental caries. Nutr. Rev..

[21] Hujoel P. (2009). Dietary carbohydrates and dental-systemic diseases. J. Dent. Res..

[22] Hujoel P.P. (2013). Vitamin D and dental caries in controlled clinical trials: Systematic review and meta-analysis. Nutr. Rev..

[23] Beckett D.M., Broadbent J.M., Loch C., Mahoney E.K., Drummond B.K., Wheeler B.J. (2022). Dental Consequences of Vitamin D Deficiency during Pregnancy and Early Infancy-An Observational Study. Int. J. Environ. Res. Public Health.

[24] Mellanby M., Pattison C.L. (1932). Remarks on the influence of a cereal-free diet rich in vitamin D and calcium on dental caries in children. Br. Med. J..

[25] Mellanby M., Pattison C.L. (1928). The Action of Vitamin D in preventing the spread and promoting the arrest of caries in children. Br. Med. J..

[26] Schroth R., Rabbani R., Loewen G., Moffatt M. (2016). Vitamin D and Dental Caries in Children. J. Dent. Res..

[27] Papas A.S., Joshi A., Belanger A.J., Kent R.L., Palmer C.A., DePaola P.F. (1995). Dietary models for root caries. Am. J. Clin. Nutr..

[28] Touger-Decker R., van Loveren C. (2003). Sugars and dental caries. Am. J. Clin. Nutr..

[29] Blake C., Bisogni C., Sobal J., Devine C., Jastran M. (2007). Classifying foods in contexts: How adults categorize foods for different eating settings. Appetite.

[30] Blake C.E., Davison K.K., Blaine R.E., Fisher J.O. (2021). Occasions, purposes, and contexts for offering snacks to preschool-aged children: Schemas of caregivers with low-income backgrounds. Appetite.

[31] Abuhaloob L., Petersen P.E. (2021). Oral Health Status and Oral Health Behaviour among 5- to 6-year-old Palestinian Schoolchildren—Towards Engagement of Parents and Schoolteachers for Oral Health through Schools. Oral. Health Prev. Dent..

[32] Borrell García C., García Miralles E., Marqués Martínez L. (2022). Association between eating behavior pattern and caries in a population of children aged 3 to 9 years in the province of Alicante. Nutr. Hosp..

[33] González-Olmo M.J., Ruiz-Guillén A., Moya-López M., Romero-Maroto M., Carrillo-Díaz M. (2022). The Influence of Parenting Styles on Eating Behavior and Caries in Their Children: A Cross-Sectional Study. Children.

[34] Mueller M., Schorle S., Vach K., Hartmann A., Zeeck A., Schlueter N. (2022). Relationship between dental experiences, oral hygiene education and self-reported oral hygiene behaviour. PLoS ONE.

[35] Nicksic N., Massie A., Byrd-Williams C., Kelder S., Sharma S., Butte N., Hoelscher D. (2018). Dietary Intake, Attitudes toward Healthy Food, and Dental Pain in Low-Income Youth. JDR Clin. Trans. Res..

[36] Pandey R., Mishra A., Chopra H., Arora V. (2018). Oral health awareness in school-going children and its significance to parent’s education level. J. Indian Soc. Pedod. Prev. Dent..

[37] Ji Y., Zhang Y., Wang Y., Chang C. (2016). Association between family factors and children’s oral health behaviors—A cross-sectional comparative study of permanent resident and migrant children in large cities in China. Comm. Dent. Oral. Epidemiol..

[38] Poutanen R., Lahti S., Tolvanen M., Hausen H. (2006). Parental influence on children’s oral health-related behavior. Acta Odontol. Scand..

[39] Eke C.B., Akaji E.A., Ukoha O.M., Muoneke V.U., Ikefuna A.N., Onwuasigwe C.N. (2015). Paediatricians’ perception about oral healthcare of children in Nigeria. BMC Oral. Health.

[40] Gereige R.S., Dhepyasuwan N., Garcia K.L., Vasan R., Serwint J.R., Bernstein H.H. (2015). Pediatric Residents’ Knowledge and Comfort With Oral Health Bright Futures Concepts: A CORNET Study. Acad. Pediatr..

[41] Hadjipanayis A., Grossman Z., del Torso S., Michailidou K., Van Esso D., Cauwels R. (2018). Oral health training, knowledge, attitudes and practices of primary care paediatricians: A European survey. Eur. J. Pediatr..

[42] Richards W., Filipponi T., Roberts-Burt V. (2014). Roberts-Burt Mind the gap! A comparison of oral health knowledge between dental, healthcare professionals and the public. Br. Dent. J..

[43] Shah K., Hunter M.L., Fairchild R.M., Morgan M.Z. (2011). A comparison of the nutritional knowledge of dental, dietetic and nutrition students. Br. Dent. J..

[44] Frame L.A. (2021). Nutrition, a Tenet of Lifestyle Medicine but Not Medicine?. Int. J. Environ. Res. Public Health.

[45] Harkin N., Johnston E., Mathews T., Guo Y., Schwartzbard A., Berger J., Gianos E. (2019). Physicians’ Dietary Knowledge, Attitudes, and Counseling Practices: The Experience of a Single Health Care Center at Changing the Landscape for Dietary Education. Am. J. Lifestyle Med..

[46] Mondala M.M., Sannidhi D. (2019). Catalysts for Change: Accelerating the Lifestyle Medicine Movement Through Professionals in Training. Am. J. Lifestyle Med..

[47] Scarborough P., Rayner M., Stockley L., Black A. (2007). Nutrition professionals’ perception of the ‘healthiness’ of individual foods. Public Health Nutr..

[48] Piñeiro R., Network O.B.O.E., Brotons C., Bulc M., Ciurana R., Drenthen T., Durrer D., Godycki-Cwirko M., Görpelioglu S., Kloppe P. (2005). Healthy diet in primary care: Views of general practitioners and nurses from Europe. Eur. J. Clin. Nutr..

[49] Parker W.-A., Steyn N.P., Levitt N.S., Lombard C.J. (2011). They think they know but do they? Misalignment of perceptions of lifestyle modification knowledge among health professionals. Public Health Nutr..

[50] Talip W.-A., Steyn N.P., Visser M., Charlton K.E., Temple N. (2003). Development and validation of a knowledge test for health professionals regarding lifestyle modification. Nutrition.

[51] Niven P., Morley B., Gascoyne C., Dixon H., McAleese A., Martin J., Wakefield M. (2022). Differences in healthiness perceptions of food and dietary patterns among the general public and nutrition experts: A cross-sectional online survey. Health Promot. J. Austr..

[52] Hancock S., Zinn C., Schofield G., Thornley S. (2020). Nutrition guidelines for dental care vs. the evidence: Is there a disconnect?. N. Z. Med. J..

[53] Gibson E.L., Wardle J., Watts C.J. (1998). Fruit and vegetable consumption, nutritional knowledge and beliefs in mothers and children. Appetite.

[54] Livingstone M.B., Robson P.J., Wallace J.M. (2004). Issues in dietary intake assessment of children and adolescents. Br. J. Nutr..

[55] Bodel S.L. The Development of a Fruit and Vegetable Liking Tool for Preschool Aged Children. Master’s Thesis.

[56] Ministry of Health (2003). NZ Food, NZ Children: Key Results of the 2002 National Children’s Survey.

[57] Hancock S., Zinn C., Schofield G. The validity and reliability of novel card sorting tasks to evaluate nutrition knowledge for general and oral health. J. Educ. Health Promot..

[58] Ministry of Health (2016). Clinical Guidelines for Weight Management in New Zealand Children and Young People.

[59] Suleiman-Martos N., García-Lara R., Martos-Cabrera M., Albendín-García L., Romero-Béjar J., la Fuente G.C.-D., Gómez-Urquiza J. (2021). Gamification for the Improvement of Diet, Nutritional Habits, and Body Composition in Children and Adolescents: A Systematic Review and Meta-Analysis. Nutrients.

[60] Walker J.L., Dix C., Hardt J., Farletti R., Littlewood R. (2022). What Do Health Professionals and Parents Want as Part of an Online Childhood Obesity Prevention Program?. Child. Obes..

[61] Statistics New Zealand (2019). Subnational Population Estimates: At 30 June 2019 (Provisional). https://www.stats.govt.nz/information-releases/subnational-population-estimates-at-30-june-2019-provisional.

[62] Hawkins M. (2021). Were warriors once low carb? Commentary on New Zealand Māori nutrition and anthropometrics over the last 150 years. J. Prim. Health Care.

[63] Schofield G., Zinn C., Rodger C. (2015). What the Fat? Fat’s IN, Sugar’s OUT.

[64] Gameau D. (2015). That Sugar Film.

[65] Monteiro C.A., Cannon G., Levy R.B., Moubarac J.-C., Louzada M.L.C., Rauber F., Khandpur N., Cediel G., Neri D., Martinez-Steele E. (2019). Ultra-processed foods: What they are and how to identify them. Public Health Nutr..

[66] Monteiro C.A., Cannon G., Moubarac J.-C., Levy R.B., Louzada M.L.C., Jaime P.C. (2018). The UN Decade of Nutrition, the NOVA food classification and the trouble with ultra-processing. Public Health Nutr..

[67] Monteiro C.A., Cannon C., Levy R., Moubarac J.-C., Jaime P. (2016). NOVA. The star shines bright. World Nutr..

[68] Campain A.C., Morgan M.V., Evans R.W., Ugoni A., Adams G.G., Conn J.A., Watson M.J. (2003). Sugar-starch combinations in food and the relationship to dental caries in low-risk adolescents. Eur. J. Oral. Sci..

[69] Achalu P., Zahid N., Sherry D.N., Chang A., Sokal-Gutierrez K. (2019). A Qualitative Study of Child Nutrition and Oral Health in El Salvador. Int. J. Environ. Res. Public Health.

[70] Martínez Steele E., Baraldi L.G., da Costa Louzada M.L., Moubarac J.-C., Mozaffarian D., Monteiro C.A. (2016). Ultra-processed foods and added sugars in the US diet: Evidence from a nationally representative cross-sectional study. BMJ Open.

[71] Kopp W. (2019). How Western Diet And Lifestyle Drive The Pandemic Of Obesity And Civilization Diseases. Diabetes Metab Syndr Obes.

[72] Miclotte L., Van de Wiele T. (2019). Food processing, gut microbiota and the globesity problem. Crit. Rev. Food Sci. Nutr..

[73] Rauber F., da Costa Louzada M.L., Steele E.M., Millett C., Monteiro C.A., Levy R.B. (2018). Ultra-Processed Food Consumption and Chronic Non-Communicable Diseases-Related Dietary Nutrient Profile in the UK (2008-2014). Nutrients.

[74] Martínez Steele E., Juul F., Neri D., Rauber F., Monteiro C.A. (2019). Dietary share of ultra-processed foods and metabolic syndrome in the US adult population. Prev. Med..

[75] Malhotra A., Schofield G., Lustig R.H. (2018). The science against sugar, alone, is insufficient in tackling the obesity and type 2 diabetes crises—We must also overcome opposition from vested interests. J. Insul. Resist..

[76] Mackay S., Mhurchu C.N., Eyles H. (2019). State of the Food Supply: New Zealand 2019.

